# Epidemic Cholera in Cincinnati

**Published:** 1832

**Authors:** Daniel Drake

**Affiliations:** Cincinnati


### WESTERN JOURNAL

OF THE

### MEDICAL AND PHYSICAL SCIENCES.

OCTOBER, NOVEMBER AND DECEMBER, 1832.

### Essays and Cases.

I.

Art. I. *Epidemic Cholera in Cincinnati.* By Daniel Drake, M. D.

### I. Outbreaking of the Disease.

From the time that Epidemic Cholera commenced at Quebec, it was an object of deep interest with me to watch its onset in Cincinnati, with a view to the great question of its mode of propagation. I propose to record the observations which I was enabled to make on this subject.

Although an inland town, of comparatively recent origin, Cincinnati is a place which has very extended and intimate relations with many others. Almost all who pass to and from the States east and west of it, and they are numerous, stop in the city. Three lines of stage coaches run perpetually between Sandusky and Cleaveland, on Lake Erie, and steam boats from Pittsburgh and Wheeling above, and New Orleans, St.

Louis, and Louisville, below, arrive and depart daily. Thus, if the Epidemic could be propagated by goods or persons, the means of introducing it were not wanting; and, as it prevailed in the basins of the Lakes and the upper Mississippi, for some time before it occurred in that of the Ohio, sources of contagion, if it be a contagious malady, were not wanting in places not more than three travelling days distance from us.

In the afternoon of the 9th of October, (1832) the steam-boat Sylph arrived at our quay, with a passenger laboring under Epidemic Cholera. He was an European, who had left Kingston, in Upper Canada, nine days before, and crossing the State of Ohio, on the canal from Cleaveland to Portsmouth, 120 miles above Cincinnati, got on board the steam boat, at that place; about which time he was seized with the disease. In a few days after this time, the Epidemic was general over the city. But was it introduced by this patient? Fortunately for the cause of truth and humanity, this question can be decisively answered in the negative.

That I may not call on the readers of this Journal, to receive my *ipse dixit*, as evidence in so important a matter, I shall at once proceed to the proofs.

The following brief histories, composed of the most authentic materials, collected at the time, published in the gazettes of the city, and never questioned, will, I think, conclusively establish the fact, that the Epidemic commenced not on the 9th of October, but on the 30th of September, nine days before.

1. John Coonse, between Race and Elm, beyond the canal, drove a waggon on Saturday, Sept. 29, in the rain, and got wet; breakfasted next morning, and was preparing to go into the country, when about 9 o'clock he was seized with vomiting and diarrhœa—these symptoms continued for several hours, with cramps, coldness of the limbs, great restlessness, and intense thirst—his mind remaining regular. In the afternoon, his pulse ceased, his face became purple, his hands sodden and blue, and at six o'clock he expired, 9 hours after the attack.

Drs. Richmond and Wm. Smith attended this patient. Dr. Staughton, of the Board of Health, saw him a little before his death. I did not see him till a few minutes afterwards. The next morning, Monday, October 1, Dr. Richmond, without having seen me, reported the case to the Board of Health as Epidemic Cholera. He and Dr. Wm. Smith, both believed at that time that such was its character.

2. John Mulholland, east end of Fifth street. Seized on Sunday night, September 30, at half past ten, with nausea and diarrhœa. In an hour had an icy coldness of the extremities, with violent spasms; ordinary contents of the stomach thrown up; from the bowels discharges at first brown, then watery and copious. Dr. Bonner, the physician called in, derived these particulars from the wife. When the Doctor arrived at 4 o'clock, A. M, six or seven hours after the disease set in, the patient presented the following symptoms: Surface of the body generally cold, clammy, and of violet color; eyes sunken, pulse gone, and respiration imperceptible—and yet the mental faculties were so unimpaired, that he asked for "25 drops of laudanum."* In a quarter of an hour afterwards he expired. I had the symptoms of this case from Dr. Bonner himself.

3. James McClintock, cook of the steam boat Huntsman, running as a regular packet between this city and Louisville, at which place there was no Cholera, was attacked on the morning of the 30th of September, with diarrhœa. After heating himself in the kitchen, he stood, for some time, on the bow of the boat, then on her downward voyage, and had a chill. Took to his bunk, when vomiting and dreadful cramps in all his extremities came on. His alvine discharges were watery; and a dark circle formed around his eyes; his pulse sunk; and his body was covered with a cold perspiration. When the boat arrived at Louisville, Dr. Powell announced him in a state of collapse; and he expired the next morning, October 1st, at 2 o'clock.

4. Susan Hation, a negress on the Green, east end of Fifth street. Taken on Sunday night, September 30th, with puking and pain in the stomach and bowels—said not to have had diarrhœa. In the language of her colored friends, 'was cramped all over.' Retained her senses to the last. Died 12 hours from the attack

5. Rachel Brown, negress, in the same house with the last. Attended by Dr. Sparks. After having had diarrhœa for some time, was attacked with violent symptoms on Tuesday night, October 2. The Doctor saw her the next night, when her extremities were cold and her pulse gone; vomited but little: had cramps when she was first taken. Died on Thursday night.

6. Mr. Carsley, in New street, near the two last, a white man, was attacked at 10 o'clock on Monday morning, October 1, with vomiting, diarrhœa, and cold sweats; severe spasms came on, and in the language of his wife, his body and limbs became 'colder than death.' Dr. Obedorff being called, gave him laudanum. On Tuesday was better; but on the night of that day, had a return of all the symptoms. Retained his senses till he expired on the night of the 4th.

7. Robert Fleming. This victim lived 12 miles from Cincinnati, in the State of Kentucky. He spent four days in the city on business, and immediately after his return home, Oct. 4, was seized with vomiting and diarrhœa in the morning. Coldness and cramps came on, and he expired before night. Seen by Dr. Riggs, of Covington.

8. Daniel Underwood, a colored man, Sixth street, east of Broadway, after attending market on Friday Morning, October 5, was seized at 9 o'clock, A. M. with griping pains, diarrhœa, and severe spasms—at half past 2 P. M. pulseless, with clammy sweats, and his skin "so cold as to feel like the marble slab of the soda fountains;" at the same time he complained of being hot; excessively restless; rather flighty or absent in his mind; voice and countenance so altered that his friends said they should not have known him. Died the same evening. Attended by Dr. Lawrence.

9. George Price, a colored man, belonging to a steamboat which had been for some days out of commission. Was at Anderson's near where the last patient lived, on Wednesday evening, Oct. 3d, and seemed well; was brought back to Anderson's on Friday evening 5th; had been seized with "cramp colic," and had "cramps all over," with cold sweats. Died soon after his return. I could not learn what physician had attended him, nor could I collect more than the above imperfect particulars for this case.

10. Ruth Hamilton, an old negress, living with a negro family, in a narrow and dirty alley, near the intersection of Sycamore and Columbia streets. Ate dinner and worked, on Thursday, October 4. Soon after dinner, which was of a common kind, was seized with "wonderful pain," and diarrhœa; vomiting of a watery fluid and cramps soon came; her skin got cold, she had "heavy sweats," complained of heat, and wanted fanning. Retained her senses to the last. Had no physician, and took no medicine. Died next morning, October 5, at 9 or 10 o'clock.

11. David Foder, a German, at the east end of Symmes' street. Had a diarrhœa for several days, but kept about. On Thursday night, October 4th, was seized with vomiting, and increase of diarrhœa. Dr. Eberle prescribed for him on Friday morning, and said he would see him again next day. The vomiting ceased, but the diarrhœa continued more or less; he was thirsty; coldness and cramps came on; his visage was blue and purple, and at 5 or 6 in the evening, he expired, retaining his senses to the last.

12. James Turner, negro, between Elm and Plumb streets, near the centre of the town, attended by Dr. Waldo, who furnished me with the facts. Went to bed well on Friday night, 6th Oct. Taken between 2 and 3, A. M. with severe diarrhœa, and violent spasms and sweats; the skin became very cold, the pulse ceased, and he expired at 6 o'clock, three hours after he was seized.

13. Mary Collins, a negress, on the Green, attended by Dr. Waldo, and visited by myself. Symptoms, diarrhœa, vomiting, cramps of the most painful kind, coldness, pulse at length imperceptible—death on the evening of the 6th.

14. Zacharias Nelson. Taken at 4 in the morning after two days of mild diarrhœa, with violent vomiting, cramps and coldness—diarrhœa increasing, with muddy and afterwards light colored discharges—in a few hours, the backs of both hands became sodden, and his nails blue; visage and gums purple; tongue and insides of the mouth cold, while he still swallowed and had his senses. Upon bleeding, the blood thick and black as tar. Expired at 8 o'clock the same evening, Oct. 6. Dr. Dart attended this patient. Dr. Marshall, saw him three hours before his death. I was with him during the last hour.

15. James Sharer, Plumb street, attended by Dr. Wiley. Attacked Saturday night, and died in four hours, with vomiting, purging and spasms. Died 7th.

16. Joseph McIntyre, near Western Row and Second streets. Taken on the night of the 7th. Seen by Dr. Shotwell. Vomiting, purging, cold sweats, universal coldness, hands sodden and deep purple—died on Sunday the 7th. Seen by Dr. Eberle and Mr. Jones members of the Board of Health.

17. Nancy Hall, negress, near the same place. Symptoms of Epidemic Cholera complicated with Syphilis, in its advanced stages. Died 7th.

18. Elizabeth Roberts, lower end of Ludlow street. Walked to meeting on Sunday night, and died on Monday the 8th. Attended by Dr. Walker—seen by Dr. Rives, Dr. Price and myself. Symptoms—diarrhœa, vomiting, coldness, spasms, sodden state of the feet and hands, pulselessness for several hours, purple gums, black spots on the whites of the eyes—smoke color of the nails, retention of the senses to the last.

19. Richard Dement, lately a citizen of Cincinnati, but at the time of his illness, an inhabitant of Covington, on the opposite side of the river. Was in the city on Saturday afternoon, the 6th of October, ate hearty, and got wet before his return. Had a chill. In the night, at 4 o'clock, was seized with dreadful vomiting and diarrhœa; threw up the ingesta unaltered, and at length had rice-water discharges from the bowels; great coldness, and severe and painful cramps supervened; his visage sunk and became purplish; his hands were shrivelled and blue about the nails; his thirst, restlessness, and desire for fresh air, were extreme, and he expired about 5 o'clock in the evening. Dr. Smith, the chairman of a Sanitary Committee, of which I shall presently speak, was one of his physicians.

20. Sears Crane, a wagoner, living in an alley between Elm and Plumb, and Front and Second streets. Got wet on Saturday Oct. 6th, and the next day felt unwell, with a loss of appetite. On Monday was seized with diarrhœa, and in the afternoon vomiting came on; all the night following he had unquenchable thirst, with cramps. His face became purple, and his sodden fingers of a smoke color. Died at 10 A. M. Tuesday 9th. Was attended by Dr. Waldo.

21. Archibald Baker, Fifth street, beyond Western Row, had a diarrhœa for some time, which greatly increased on Wednesday Oct. 3d. He subsequently had cramps in the stomach and legs, with coldness of the latter, and still greater coldness of the tongue, which with his lips became purple. His discharges were watery, his respiration gasping, and his pulse became imperceptible several hours before his death. He died on Monday morning, Oct. 8th. Attended by Dr. Richmond.

Thus, between the 30th of September and the 8th of October, 21 fatal cases of Epidemic Cholera occurred in Cincinnati, before the arrival of the boat, which brought the patient from Portsmouth. It was not, however, till after the occurrence of that case, that the Board of Health admitted the existence of the Epidemic in the city! Had they been ignorant of these cases, their failure to announce the existence of the Epidemic before the 10th of October, could not have been censured. But this was not the fact. On Saturday, the 6th of October, I addressed a note to a member of the Board, in which I gave him the names of ten of the victims mentioned above, and assured him that they had died of Epidemic Cholera within the week; the next day, I added another, and the succeding day detailed, to a different member, the symptoms of another, who had just expired.

My *unofficial* note, (for as the patients whose cases I reported were not mine, I was not required by the ordinances of the council to report them,) was referred to a committee of the Board, who, as I ascertained, intended to report, that they were *not* cases of Epidemic Cholera; and on the same day, that this reference was made, the Board sought an interview with Gen. Scott, then in the city, to request of him not to suffer the troops, who were returning from the Indian war, on the upper Mississippi, and had been affected with Cholera, to land at this place, lest they should communicate the disease.

Regarding it as important to the safety of the people, that they should be apprized, without delay, of the existence of the Epidemic, and thoroughly convinced that it had been upon us for a week, I spoke publicly of the fact, immediately after my communication to the Board. The next morning, Sunday, I went still further. As a large proportion of those who had died were colored people, and the whole of that community were profoundly ignorant of the character of the disease and the means of prevention, I determined to address them on the subject. This I did in the afternoon of that day, in one of their largest churches; and have reason to believe that some good resulted from it, notwithstanding it was made a subject of derision, during the hour of its delivery, by the chairman of the committee, of which I have just spoken.

In this address, which was attended by several respectable and benevolent citizens, I asserted the undoubted existence of the Epidemic; but still the notification was so partial, that the great mass of our population were totally unconscious of the necessity of an immediate change in some of their habits, and of the importauce of attending strictly to the first symptoms of any disorder which might assail them. Under these circumstances, I felt at liberty to make a more public enunciation of what had fallen under my notice; and accordingly, in the afternoon of the same day, sent a communication to the offices of the different daily gazettes.

The distribution of the papers on the following morning, excited a deep sensation throughout the city. Many believed in the reality of what was stated, and censured the Board of Health; but more were incredulous, or affected to be, and expressed their reprobation of the author of the notice, in terms of unmeasured obloquy:—A fact that will account, and I hope be a sufficient apology for, this circumstantial narrative.

The Board of Health, not less than many of our citizens, were thrown into a state of excitement by this publication; and immediately adopted a measure, which they presumed would relieve them from the reproach of unfaithfulness. This was to appoint and organize a "Sanatory Committee" of seven physicians, two of whom were members of their own body. These gentlemen were assembled *forthwith*, and proceeded to act *instanter*. They did not, however, go in quest of the information, necessary to a correct decision, nor send to me for the history of the cases I had reported; but, in the very hour of their appointment and convocation, drew up and subscribed the following certificate.

"*Board of Health, Cincinnati, October* 8, 1832.

The following report was made this day to the Board of Health, which being read was ordered to be published.

*To the Board of Health:*

The sanatory committee, acting under the authority of the Board of Health, report: That they have inquired into the circumstances and character of the cases which have been published as instances of Epidemic, Malignant, or Asiatic Cholera, together with some other cases of a like character, and from their own personal knowledge of many of those cases, and from information carefully collected in relation to the rest, they are convinced that these diseases have arisen from the usual exciting causes of bowel affections, or common cholera morbus, and that several of them have not been cases of cholera of any kind. (Signed)

*Jesse Smith, M. D., Jedediah Cobb, M. D., V. C. Marshall, M. D., John Moorhead. M. D., Abel Slayback, John Eberle, M. D., J. M. Staughton, M. D.*

Cincinnati, October 8, 1832.

The Board of Health assure their fellow citizens they will continue to investigate and examine into the character of all cases which come under their notice, and when any case shall be found to justify a belief that the epidemic cholera exists in this city, they will forthwith report the same to the public."

This document appeared in the gazettes, October 9th, the next morning after my publication, and was expected by the Board to quiet the public mind. The odds of seven to one, seemed fearful, but the contest was for a few hours only. Before night, the Epidemic had itself become umpire, and established the alarming fact of its existence in every part of the city. The papers of the next morning, October 10, contained a report by the Board of Health, of four deaths from malignant Cholera; and the Sanatory Committee were never heard of again throughout the Epidemic!

For the moment, however, their certificate placed me on the horns of a dilemma. I was the oldest physician of the city, and had resided in it from the time it was an unsightly group of log cabins; I had written much for popular use, in its newspapers, through the summer, on the means of preventing the Epidemic; and had compiled and published a volume, on the same subject, for the profession in the valley of the Mississippi; but still, all who believed the Sanatory Committee, must believe me either ignorant of the characteristic symptoms of Epidemic Cholera; or disposed, wantonly, and wickedly to sound a false alarm.

The man of sensibility, wherever he may be, who can imagine himself placed in this predicament, will, I am sure, be inclined to pardon these details; and think it strange, that this committee should have felt themselves at liberty, or considered it safe for their own character, thus precipitately to impugn the accuracy, or the integrity, of a professional brother; but the fact is, that most of them were chosen, not for their *superior* professional standing, but for their known and ancient hostility to the humble individual, upon whose professional report they were called to sit in judgment. They were convoked, to gainsay his publication; and did not disappoint, if they failed to extricate, their employers. The chairman of the committee, moreover, going a little beyond the Board, and, the City Council, had, previously, declared his belief in the contagion of Cholera; and, although, but the day before, he had closed the eyes of Mr. Dement, who exhibited the characteristic symptoms of that malady, in an exquisite degree; still, he would not admit the existence of the Epidemic, till, on the succeeding day, a man arrived in a steam boat, with the disease upon him!

That the committee, in making up their certificate took counsel from their own feelings and the wishes of the Board of Health, instead of the facts which should have guided their inquiries and controlled their desires, I am compelled to believe; for they acted without the information necessary to a correct decision; and one of them, the week before, had declared his conviction that the Epidemic influence was then upon us; another, on the very day that my publication was sent to the press, traced out the different symptoms of two patients then in a state of collapse, and pronounced them identical with those detailed in the books; and a third has since admitted, that he believed the cases I had reported to be Epidemic Cholera, at the time he signed the certificate! I take no pleasure in exposing these circumstances; but having through life made it a rule to correct misrepresentations of my character, either moral or professional, as soon as they were uttered, I am constrained to record this correction; and having done so, shall leave with pleasure, that which I approached with very different feelings.

There is, however, a much higher reason than one merely personal, for reviewing the proceedings of the Board of Health and their medical advisers. As the former was established by the City Council, under a law of the State, all its acts, including those of the latter, were official, and its publications might be referred to by the historians of the Epidemic, as authentic and credible. Now, on the 9th of October, these two public bodies, inform the world, that up to *that* time, there had been no Malignant Cholera in the city; but on the evening of that day, a steam boat passenger, emigrating from Canada, arrives with the disease upon him; and, expiring the next morning, is reported by the same authorities, as having died of that malady. Here, then, is a state of fact, which, considered apart from any other, would call for the conclusion, that the Epidemic had been actually introduced from the St. Lawrence—than which, nothing could be more foreign from the truth. The details which I gave, at the time, in the newspapers of the city, which were never contradicted or even doubted by its whole reading population, and, which are now presented to the profession, will serve, I trust, to preserve the future medical historian from so great an error.

Such is the history of the onset of Epidemic Cholera in Cincinnati. That it was not introduced from abroad, either by persons or things, is now universally believed; and, I may add, during its whole continuance, not a single fact occurred, that went in the slightest degree to establish its contagious character. In a few cases, it is true, several members of a family were swept off in rapid and frightful succession; but the physicians, nurses, and visiters of these very families, escaped it. Instances of this kind are to be explained in part, by a reference to the similarity of constitution and the strong common susceptibility, existing in some families; and by the alarm, confusion and neglect of early symptoms, which were the necessary consequence of more than one member of a family, happening to be taken down at the same time.

Still it is undeniable, that facts have transpired (some of which will be found in the present number of this Journal) which seem to support the opinion that Epidemic Cholera *sometimes* spreads by contagion. I can only say, that admitting this to be true; the disease *may* originate independently of contagion. How many maladies conform to this law has not yet been ascertained; but canine madness is an example which cannot be questioned. Measles, scarlet fever, and typhus are more doubtful in this respect; but there are many facts which seem to indicate, that they are both epidemic and contagious. If, however, this be the case with Cholera, there can be no doubt that its contagious power is extremely feeble, and that it, generally, almost always indeed, depends on some unknown epidemic distemperature of the atmosphere. Its spread along navigable water courses, has been remarked from its origin in Asia, where, however, it is said to have extended, along the rivers that were not navigated, not less than those which were. Opportunities for observations on this latter point, do not exist in the United States, where every stream deep enough to float a steam boat is navigated; but of the tendency of the disease to appear first in the principal commercial towns and to stretch along our great rivers there can be no doubt. The facts on this point which relate to the Ohio and Mississippi, I shall mention presently.

It being definitively established that the Epidemic did not reach Cincinnati by contagious propagation, to what cause should it be ascribed? This is a question, which no one can venture to answer. The facts furnished by its outbreak here, are altogether of a negative character; but prove, conclusively, that it did not reach us by contagion. They even prove more. They show, in a degree equally conclusive, that it did not originate from the sensible states of the atmosphere, for, without going into meteorological details, it may be safely affirmed, that there was nothing in the temperature, winds or weather, of September and October, that was in any degree peculiar, or different from ordinary autumns. Nor had miasmatic localities any apparent agency in its production; for it appeared in all parts of the city, high and low, thickly and thinly peopled, clean and dirty, nearly at the same time. Finally, it was not the offspring of a deleterious meteoration, gradually advancing upon us, and indicating its approach by its effects on human life as it passed along, for all the towns and country between the shores of Lake Erie and the banks of the Ohio river, an average distance of 250 miles were free from it, when it suddenly invaded, and rapidly pervaded, every part of our city. It appeared, however, nearly at the same time at Madison, 80, and at Louisville, 150 miles further down the Ohio; and at St. Louis, on the Mississippi, about 400 miles to the west and 175 miles above the confluence of the two rivers. Within a fortnight from its attack on these places, it showed itself at Maysville, Wheeling, and several other towns on the Ohio, above and below Cincinnati, leaving many places untouched. Thus, after reigning from June to October, in some part of the valley of the St. Lawrence, and its parent lakes, it suddenly appeared and spread up and down that of the Ohio, leaving the country between, untouched. From the southern shores of this river it advanced into the interior of Kentucky, reaching Frankfort and Lexington, near the latter end of October, and about the same time, commenced its ravages in New Orleans; while much, indeed, most of the extensive intervening country, between this and that city, like the State of Ohio up to this time, Nov. 30th, remains unaffected.

But we must not suppose, that the malady has appeared along all our considerable water courses. The valleys of the Muskingum, Sciota, and Great Miami, traversing the southern half of the state, from north to south, have not been ravaged; nor have the people who live on the banks of the Ohio and Erie Canal, or the Cincinnati and Dayton Canal, been affected. The facts relative to the last, deserve a moment's notice. This canal runs for 20 miles back for Cincinnati, in the broad and deep alluvial valley of Mill Creek, when entering that of the Great Miami, having the same geological character, it continues in the vicinity of that river, to Dayton; passing through Hamilton and Middletown, besides a number of villages. Near the route of the canal lies the great road, along which are a line of daily stages to Dayton, and two to Hamilton. Throughout the whole reign of the Epidemic in Cincinnati, canal boats for Dayton, a distance of sixty miles, and for all the intervening towns, were departing daily with goods and passengers of all kinds; and the stage coaches were likewise filled as usual. But, still, the place just named, and indeed the whole valley of the Great Miami, remain to this hour uninfected. Now, if the Fpidemic can be spread either by men or merchandize, why was it not carried back, from Cincinnati, during the 30 days in which it prevailed? If it could be brought to this place, without our knowing whence or by whom, why should it not have been carried up the valley of the Great Miami, traversed as it was, by multitudes leaving our city every day? If it could be freighted from Cincinnati to New Orleans, in a steamboat, why was it not carried from the same city, to Dayton, in a canal boat; especially when the latter voyage occupies only a tenth part of the time of the former? These are questions which I think, the contagionists ought to consider and answer candidly.

In the last number of this Journal, I gave a brief history of the epidemic constitution of the summer season, which had just then expired. A perusal of that sketch cannot fail to impress the reader with the opinion, that in the months of June and July, we had, at this place, a distemperature of the general atmosphere, differing only in degree, from that which prevailed on the St. Lawrence. A state of unusual morbid irritability, in the digestive organs, with numerous cases of mild or profuse diarrhœa, was almost universal through this region, particularly in the towns and cities; and many fatal cases of cholera, attended, indeed, with symptoms strongly characteristic of the Epidemic, occurred in the practice of different physicians; and suggested in their minds an apprehension, that the great visitation had actually commenced. With the departure of summer, this constitution subsided; and September was unmarked by any peculiarity in its diseases. With October the former state of things arose anew. The Epidemic, in its full and awful power was now upon us. Diarrhœas and morbid sensibility of the stomach and bowels were reproduced throughout our whole population; and fatal cases of cholera occurred by hundreds. Thus we have, in reality, experienced two invasions, one in June, of a mild and ambiguous character, in connexion with our endemic cholera morbus, and cholera infantum; the other, in the last week of September, in connexion with our autumnal fevers; or rather as a substitute for them, as they have, I think, been less prevalent this Fall, than usual. These facts, for such I esteem them, seem to me worthy of being recorded.

### II. Progress, Duration, Eclectic Character and Mortality of the Disease.

The Epidemic, as I have just observed, may be said to have commenced with the month of October. During the first week, scattering cases, of a most fatal character, were almost all that occurred. The second week brought forth a frightful number of mortal, and a far greater number still, of mild cases. Through the third week the mortality was increasing; its maximum for a single day, was from noon of the 19th to the same hour of the 20th, when 42 deaths were reported to the Board of Health, which doubtless did not embrace the whole. Early in the fourth week, it began to decline, and by the end of the month was nearly extinct—no deaths being reported by the Board after the 4th of November. It prevailed as a *pervading* and fatal epidemic about two weeks, but its whole duration was six.

Much has been observed and written on the eclectic propensity of this malady. In Cincinnati, this propensity was, I think, less manifest, than it seems to have been, elsewhere. The burden of mortality, certainly fell on the laboring classes, in low or middling circumstances; but these, it may be observed, make up the mass of the population; and, with due allowance for their neglect of the early and curable stages of the disease, it may be doubted, whether they were more *predisposed*, than the wealthy and more comfortable portions of the community. Of those who are sunk into abject poverty, with its noisome concomitants, filth and starvation, Cincinnati happily has but few; of these, a portion died, but many survived; and there does not seem to be any sufficient ground for affirming, that this degraded class suffered in a peculiar degree; but on this point I may be mistaken.

With respect to the intemperate, considered apart from the conditions to which I have just referred, I can speak with greater confidence. It is generally admitted, that contrary to all expectation, they were not more liable, than the temperate. No citizen of Cincinnati who will make an estimate of the proportional mortality of this pitiable class of the community, as far as his own personal knowledge can go, will, I think, dissent from what I have here stated. To what this exemption, in *our* city, so great when compared with many others, should be ascribed, it is difficult, perhaps, to say. Before the Epidemic set in, the Cincinnati Medical Society, drew up and published a paper of advice and instructions to the people, in which, referring to the use of ardent spirits, they advised, *that the temperate should not resort to drink as a means of warding off the disease, nor the intemperate refrain entirely, but only avoid drunkenness.* This advice was attacked in the newspapers by an anonymous writer, and pronounced unscientific and immoral. His strictures, provoked replies; and thus the whole matter became a current topic of popular interest and discussion, till it spread through all classes. How far this admonition was acted upon, either by the temperate or the intemperate, and contributed to the preservation of the latter, I am unable to say; but can assign no other cause for the escape of so large a proportion. Of the strictly sober and orderly members of society, it is quite certain, that not a few perished; while habitual drunkenness on either side, remained exempt, or were but slightly affected.

I expect to be censured for *publishing* this fact. But I am writing a *medical history*, not a *temperance address.* From the latter, such a statement might properly be excluded; but the cause of scientific truth suffers from the suppression not less than the perversion of facts. There are obligations to science, as well as morality; and they can never, in fact, be incompatible. One of the physicians of the north of England, in writing of the epidemic to a friend, declares, that it affected the temperate and intemperate equally; but, adds, that for the interests of sobriety, it might be well enough to let the current opinion go uncontradicted. However, we "should not sin that grace may abound," and until the end will justify the means, we ought neither to suppress nor exaggerate facts in our efforts to prevent drunkenness. Moreover, I cannot attach much importance to the argument against that vice, which is furnished by the prevailing opinion, of the liability ot the intemperate to Cholera. To employ a slang phrase, suggested by the subject, as soon as the epidemic has reigned its 30 days and passed away, they who had held up, are sure to "treat resolution," and before the end of the year, ingurgitate the annual, average quantity. Those who war against intemperance, with its concomitant vices, may find in their magazines an abundance of weapons; without laboring to wield an occasional and transient epidemic against the hydra headed monster, whose ravages on society, are equally relentless in the midst of all events and through all seasons.

Our black population were unquestionably more liable to the disease, than the whites; and this, I apprehend, from national susceptibility; for it was by no means confined to the dissolute and indigent, but carried off a due proportion of those in comfortable circumstances and of respectable standing.

In its milder grades, the epidemic affected all ages and both sexes of whites, nearly in the same degree; but proved more fatal to adults than children; and to men than women. It is doubtful how far a difference of constitution contributed to this difference in mortality between the two sexes; for as the occupations of man carry them more into the open air and expose them oftener to inclement weather, than those of women, we may ascribe a part, at least, of their greater sufferings to those causes—so apt to excite and aggravate the disease.

The total number of deaths from the Epidemic, will never, perhaps, be accurately known. Mr. Morrison, the clerk of the township, after availing himself of all official returns, and every other practicable source of information, estimates the number, from the 24th of September to the 20th of November, a period of eight weeks, at 571; which prnbably is not far from the truth. Supposing 26 of these to have been among strangers landing at the quay, we have a remainder of 545, for the mortality of the city. Of these 45 were negroes. Estimating our population at 30,000, of which 1500 may be negroes, and deducting them and the whites who fled to the country from the whole, we may assume 25,000 as the white population of the city and its liberties, during the Epidemic. Of this number 500 died, or one out of 50, a loss of two per cent., which is greater than that of most other places.

Among the whites the proportion of children who fell victims, was as 22 to 478; or something less than a 22d part.

I have, already said, that more males than females were lost. Excluding chtldren whose sexes were not noted, the deaths among our adult white population, were 478, of whom 330 were males, and 148 females, being nearly as two and twenty-three hundredths to one. To take a different view of the relative mortality of the two sexes—the loss out of our male population was nearly one in 40, or more than two and a half per cent.—that sustained by our female population, was nearly one in every 80, or one and a quarter per cent.—giving a proproportion of mortality between the sexes as two to one.

I have likewise said, that our colored population suffered more severely than the white. Of the 545 who died in Cincinnati, and were of the city, 45 were negroes; although our black population does not exceed 1500. The ratio of its loss was therefore three per cent.—or compared to that of the whites as one and a half to one.

### III. Stages, Varieties, and Complications of the Disease.

It is a notorious fact, that in the temperate zone, Malignant Cholera has been every where preceded, or accompanied by, an epidemic diarrhœa. Now, these two affections either depend on the same, or on different causes. If the latter, why do they arise and subside together? Why does a neglected diarrhœa so often terminate in cholera? Why is the latter, in all individual cases, preceded by the former? Why does this, so often follow on that, in persons whose lives are saved? And why are the intestinal secretions so much alike in the two? Finally, if they have not a common origin, two distinct atmospheric poisons are generated at the same time, and entering into combination, agree to traverse the globe in the same direction, and make a simultaneous, but separate, attack upon the health and life of its inhabitants—occasionally coming to each other's aid in the work of destruction!

It seems to me unnecessary to multiply arguments to prove, that these two affections do *not* depend on distinct, but a common cause; and are, in fact, only different grades of the same disease. If this be true, it follows, that every kind and degree of indisposition, from the slightest affection of the stomach and bowels or of any other organ up to the most malignant—which depends on the atmospheric distemperature, is, in reality, a case of *the Epidemic*, though not of cholera morbus.

Even mortal cases have not, however, always presented the phenomena of that affection; while the milder, exhibit them in every degree and relative proportion to each other. With these facts before us, may we not wonder, that Boards of Health and governments, have been, every where, requiring medical men to report their *cases* of Cholera; at the same time, not expecting to have all cases of indisposition arising from the epidemic influence, included. I can perceive no truth or value in such reports. It is the deaths, only, that should be registered The relation of these to the population of a place may be ascertained; (and for this purpose, as well as to erect Cholera hospitals for the dying, boards of health are useful;) but the proportion of deaths to the number *indisposed* from the epidemic influence, is a problem which no board of health from Astrachan to New Orleans, has been able to solve. This ancient phrase, cholera morbus, suggesting, as it always does, a particular concourse of symptoms, is not, in fact, a suitable appellation, for an epidemic, which, in the majority of cases, does not exhibit that concourse; and should, though it cannot, be discarded. Its employment has led to the absurdity, of calling the milder forms and stages of a disease, *premonitory* of the more violent; and contributed powerfully and fatally to divert the minds of both physicians and people, from the former to the latter, when in fact the *reverse should have happened.* From this single error, millions of human beings have perished. I grant, that almost all who have written on the Epidemic, have spoken of the importance of attending to the disease in its first stage, which they have still, however, regarded as a mere state of premonition; of which they might treat in a short compass; while their greatest power has been reserved to pourtray the striking phenomena, morbid appearances, and complicated treatment of that stage, which in spite of all human genius, and an experisnce copiously accumulated, from the banks of the Ganges to the delta of the Mississippi, continues, in the majority of cases, to prove fatal; and which, I venture humbly to predict, would still prove fatal, were this pestilence again to overspread the earth. Had the horrors of this mortal stage not taken possession of the feelings and imagination of the world; and had physicians, the clergy, learned societies, boards of health and sanatory committees, governments, and benevolent individuals, every where, directed their chief attention upon the first and curable, instead of the last and incurable, stages of the disease; and labored to instruct the minds, and awaken the fears of the people, (in advance of the Epidemic) on the subject of its first symptoms, I conscientiously believe, that one-half its mortality would have been averted.

To Dr. Kirk, of Greenock in Scotland, whose recent pamphlet on the Epidemic, was designed to prove, that a curable, *always* precedes the incurable stage, the interests of humanity are under the deepest obligations, though, his book has produced, I fear, but little effect; and the remark made by several medical gentlemen, in Canada, to Drs. DeKay and Rhinelander, that if a physician were sent for at the same moment, to see two patients, one of whom was slightly the other severely ill with the Epidemic, he should go first to the former, whom he might save, is entitled to universal approbation, and ought to be adopted as the rule of professional action.

I had not seen their Report containing this sound and precious advice, when the Epidemic made its advent at this place; but the experience of the few first days brought me irresistably to the same conclusion; and, I believe, produced a similar conviction, in the minds of many others. From this moment, I felt it a duty by every possible effort, to carry this momentous truth home to the understanding and feelings of the whole community; and, accordingly, inserted in the newspapers, and disseminated in the form of handbills, those solemn warnings and exhortations to the people, which brought upon me the sarcasms of those who should have been employed in the same duty. In reply to the whole, I shall only say, that the consciousness of having thus saved the life of a single human being, weighs more with me, than all the sneers, to which my humble, but earnest exertions gave origin.

The symptoms of the Epidemic presented in Cincinnati, no peculiarity worthy of notice. In its early periods, diarrhœa was the prominent affection; and the discharges were, *generally*, such as indicate a deficient biliary secretion with an increase of that from the mucous membrane. In many instances it was attended with abdominal pain, in others not. In some cases the diarrhœa was replaced by constipation; a condition, which a number of persons labored to promote or suffered to continue, under a mistaken idea, of its preservative influence. Loss of appetite, nausea, and moderate vomiting, occasionally attended the early periods of the complaint; but more commonly, the stomach was much less affected in these stages, than the bowels. The skin, like the liver, failed in its functions, from the beginning, and was prone to become dry, cool and husky. Some persons had an excited and feverish circulation; but in the greater number, the pulse was reduced in energy, and afforded no evidence of inflammatory orgasm. The muscular system was generally reduced in its powers, and more or less, affected with cramps or slight spasms, particularly in the lower extremities, symptoms which frequently affected those who labored under no obvious disorder in the digestive organs. Multitudes had only these symptoms; and, when attentive to their modes of living, and careful, in the more violent cases, to seek early medical advice, escaped the more formidable accession of symptoms, which indicated a dangerous condition. This access was sometimes so sudden, that the person, occupied abroad, would be unable to walk home, and even sink down on the ground from debility. Such instances, not, however, very numerous, spread great terror through the city; as the individuals were, generally, supposed to have been previously in good health. I believe, however, that without a single exception, they had labored under a previous diarrhœa, which they regarded as something distinct from Cholera, or as a stage of that disease, which might be safely neglected; and therefore continued on their feet in the open air, the very worst condition under which they could have kept themselves.

The transition of the disease, from a mild to a malignant state, was often spontaneous; but more frequently could be traced up to irregularities of diet or drinks, great exertion, exposure to the night air, alarm or getting wet.

The symptoms which attended the exasperation of the malady, were, generally, increase of diarrhœa—the excretions becoming more serous, and assuming the appearance of rice-water, gruel, or milk and water; vomiting; great distress in the pit of the stomach or in the bowels; thirst; a hurried state of the circulation, with a pulse, sometimes augmented, but oftener reduced in force; great muscular debility; restlessness; anxiety; and, in general, more or less chilliness. When these symptoms supervened, the patient was, I believe, always lost, if not preserved by medical skill. This, however, was, in most cases, called to his aid, for the patient, *now*, believed himself affected with Cholera, and took that alarm, which, coming upon him at an earlier period, might have prevented a concourse of symptoms, that too often proved fatal, notwithstanding a resort to the aid of our ablest pratitioners. Even in this condition, however, it happened (within my own knowledge) that from the multitude of strangers of the poorer and more ignorant classes, at all times in Cincinnati, some were unattended by any physician, and expired without having been made the subjects of any regular treatment! No part of this blame should, I think, be attached to the Board of Health or the Trustees of the Township, who, as far as I saw or heard, were humane and diligent in discharge of their duties throughout the Epidemic; nor should it fall on the medical gentlemen of the city, who were equally indefatigable in their labors, and gave promptly to the poor, both in advice and medicines, whenever called upon for either. And here I must be permitted to indulge in a sentiment of professional pride, while I claim for those who pursue the practice of medicine, while pestilential diseases prevail, a degree of praise which I think cannot justly be claimed for any other body of men in society. Their personal exposure and fatigue are incessant, and the night brings them but little rest; their attention is distracted by opposing importunities; their understandings perplexed, and their feelings tortured; their earnings, it is true, are increased, but their charities are, also, augmented; and these are not unfrequently dispensed in money, as well as visits, advice and medicines. On the whole, apart from all apprehension of personal danger, no physician can desire to encounter a pestilence a second time; and no community thus visited, should withhold from a profession which bears the shield of Minerva, and intercepts the arrows of death, the tribute of its gratitude.

The stage of Cholera, to which I have just referred, when not arrested by art, passed into that of general prostration, or collapse, as it has been figuratively called. The vomiting, if violent before, now often abated, and sometimes ceased altogether; the diarrhœa became less profuse, or stopped entirely; the spasms and cramps in some cases became more violent, but in others subsided; the same thing was true of the jactation or restlessness, which in some patients continued to increase, while in a greater number, perhaps, it gave place to a mild degree of coma; the difficulty of breathing, and the desire for fresh air, were in most instances distressing. In all cases, the pulse sunk rapidly, becoming weak, thready, vermicular, and at length imperceptible, though the patient might still live on for hours. With the faultering of the pulse, the heart also failed; and, while the patient was still conscious, and obedient to all that was said to him, that organ would contract so feebly, that neither its sound nor impulse could be perceived, by the ear applied to the chest. Coldness of the surface, particularly of the extremities, the mouth and tongue, was among the symptoms of the stage we are now considering. Suffering no diminution, it often became icy, long before the patient expired; and was, generally, attended with a profuse discharge of perspiration, which of course, felt cold at the moment of its transudation through the skin. While the secretions from the gastro-intestinal mucous membrane, and the external surface of the body were thus successively increased, that from the kidneys, was diminished or entirely suspended. I have no doubt, from the complexion which the patient assumed, that the pulmonary secretion of carbon, was likewise much reduced; but know of no direct experiments having been made to ascertain the fact. The morbid complexion, was some shade of purple or blue, inclining to smoke color. The former exhibited, chiefly, by the face, the gums, and the epithelium of the lips, the latter by the toes and fingers. These discolorations, which very much resembled those attendent on the last stages of acute bronchitis, continued after death, and might easily be distinguished from the hyperæmias, formed at that time. In one case which fell under my observation, the adnata of the eyes, exhibited large, irregular spots, as black as charcoal. The parboiled and sodken appearance of the hands and feet, described by all the historians of the Epidemic, was seldom absent. Finally, the intellectual functions, although in most cases reduced in energy, and often somewhat irregular, were, generally, so unimpaired, that the patient would attempt to project his tongue or swallow, when bidden, and often manifest an interest in the means of cure, till within a few minutes of his decease. This event was sometimes preceded by violent gaspings and agitations; but other cases presented a departure so calm and resigned, that the moment of dissolution could scarcely be perceived.

The duration of the state of collapse was various. When the patient was not resuscitated by art, he sunk rapidly; but, in some instances, his resuscitation was prompt and perfect, and his recovery speedy. In others it was partial and imperfect, and he would live on, sinking and rising, like a drowning man, for two or three days, and then expire, or slowly recover. Of those who fell into a deep and decided collapse, but few seem to have been saved.

It is impossible to say what proportion had consecutive fever. When it occurred, it was generally attended with extreme irritability of the stomach, and often with a manifest affection of the brain, accompanied, in some cases, with an engorged and purulent state of the eyes.

Considering that the Epidemic invaded us, the season when the autumnal intermittent and remittent fevers prevail, I cannot learn, that so many cases commenced and terminated with that febrile type, as might have been expected, though the number was not a few. Dr. Finley has communicated to me a case, commencing as Cholera and passing into the stage of collapse, which was evidently modified, by the endemic, autumnal distemperature; for after being preceded by a moderate degree of reaction, the paroxysm of collapse, unattended, however, with chilliness, recurred for three successive mornings, succeded each time by reaction in the afternoon, The patient, in obedience to his own instincts was stimulated powerfully and recovered. I was called into consultation, by my late pupil Dr. Shotwell, in the first week of the Epidemic, in consequence of a similar transition. His patient, a young man, was recovering from an attack of intermittent fever, when diarrhœa supervened; his circulation began to fail, his extremities became cold, and his hands sodden. In this condition he struggled on for several days, his pulse being nearly or quite imperceptible for a day or two before his death; while his intellectual functions were but little impaired. In consultation with Dr. Henry, I saw another case, which presented nearly the same mode of termination. An infant, after laboring under a malady which presented the phenomena of remittent fever, seemed to become convalescent; when its pulse began to sink, and at length became imperceptible, with other symptoms, characteristic of the stage of collapse, which continued till it expired.

A number of cases occurred under the denomination of autumnal fever, but attended, with diarrhœa, instead of the constipation usual in that fever, with serous alvine discharges and wandering cramps.

Dr. Woodward has obligingly favored me with the notes of a case of Cholera complicated with scarlatina. The patient, a female child, aged four years, was seized with fever, swelling of the tonsels, difficult deglutition, diffused inflammation of the fauces, and *vomiting*. In a few hours a *diarrhœa* came on attended with watery discharges, which, at length, assumed the *rice-water* appearance. In a couple of days, a scarlet rash showed itself on the lower part of the abdomen, which soon assumed a mahogany color, and before the little patient expired, this complexion became general.

The very first case of the Epidemic which occurred in my practice, in September, was attended with copious and alarming uterine hæmorrhage, during nearly the whole period of the vomiting and diarrhœa. The patient's pulse became nearly imperceptible, her skin cold and damp, and her lips purple; but contrary to expectation, she recovered.

Dr. Henry also met with two or three cases of hæmorrhage, or excessive menstruation, in connexion with Cholera; but whether they occurred in the practice of other gentlemen I cannot say.

Towards the decline of the Epidemic, many cases were complicated with, or terminated in dysentery.

As might be supposed, hysteria was a frequent occurrence throughout the whole Epidemic. In many instances it was excited by apprehension of the cholera, in others by the cause of that malady, more commonly by both. The excessively reduced diet, moreover, to which some persons subjected themselves, predisposed them to nervous irritations. In some cases, the paroxysm of hysteria subsided, without being followed by Cholera; but in others that malady supervened. I met with one lady in whom the Epidemic commenced as hysteria, with extreme anxiety of mind, following on a sudden death in the family from the prevailing disease. No vomiting existed, and the bowels were rather costive. The nervous irritation, with some degree of fever, continued for two days, when vomiting, cramps, and coldness, suddenly supervened; and the patient, with blue and sodden hands, expired in about eight hours. Hence it was important, for the physician to be on his guard, in reference to hysterical symptoms.

An equal degree of vigilance was, in fact necessary in regard to every kind of indisposition which occurred, for any departure from health, by whatever cause produced, was apt to terminate in Cholera. I met with one case, in which the patient, taken down with an inflammatory affection of the brain, to which he was liable, had slight *cramps* from the beginning; and ten or twelve days from the time of attack, after he had lost more than sixty ounces of blood, many additional symptoms of Cholera supervened, and contributed to prolong his confinement.

In its disposition to bend itself with, or modify other maladies—to terminate in them, and be excited by them—the Cholera but conformed itself to the laws which govern other epidemic diseases. Dr. Rush, among the more modern writers, has spoken exphatically on the importance of keeping these facts constantly in view, when a particular atmospheric distemperature is prevalent. The following anecdote will show, that this distinguished physician, observed his own precepts. During the Yellow Fever of 1793, on entering the suburbs of Philadelphia from the country, he was stopped to see a man, who had just got his leg broke; not practising surgery, he directed the friends to send for a surgeon, and contented himself with prescribing his favorite "ten and ten" dose of calomel and jalap. The by-standers thought him deranged; but the Doctor knew from experience, that such a casualty was likely to awaken in the system of the patient, the fever to which they were all so strongly predisposed, and he prescribed accordingly.

I have stated a case which presented an actual complication of Cholera with scarlatina. I come now to say, that the latter affection, has been, and still is, a coadjutor of the former, in the work of death throughout the valley of the Ohio. In the winter of 1830–31, it was extremely prevalent, and fatal in Pittsburg; and ever since, it has been prevailing in different parts of Ohio and Kentucky—chiefly in the country, and smaller towns. Scattering, and generally mild cases only, have occurred in Cincinnati; but in Lexington, it has within the last few months, been fatal to many children. It would almost seem, that there is some connexion or *understanding* between this epidemic and Cholera. I believe, that as yet, they have not both been decidedly prevalent in the same place. Pittsburgh, which was so fatally visited by scarlatina, has had very little Cholera; nearly the same remark is true of Lexington. The State of Ohio, moreover, has been extensively visited by scarlatina; but remained almost exempt from Cholera, except in Cincinnati. That city, on the other hand, has been spared the scarlatina, but sorely visited with Cholera. The former disease has come even to its limits without entering to any formidable degree. In the village of Montgomery, 17 miles north of the city, it was extremely prevalent during the preceding summer, when scarcely a case occurred here. Dr. Branch, a respectable young physician of that place, has given me an account of nearly 30 cases, which presented themselves in his own practice.

Since writing the above, I have been informed by Dr. Hildreth, an intelligent student of medicine, that, the scarlatina has prevailed for some time past, in the valley of the Muskingum, where little or none of the Epidemic Cholera has shown itself. From Dr. Johnson, of McConnellsville, on the bank of that river, he learned, that the scarlatina had been, during the past summer, extremely prevalent at that place; and was attended with such an irritable state of the bowels, and so great a tendency to diarrhœa, as to preclude the use of all cathartics except calomel, and suggest to his mind, that the scarlatina and Cholera influences were actually combined; the former, however, being predominant.

In the last number of this Journal, I have mentioned, that an influenza of a decidedly violent type, traversed the western country in November, December and January, 1831–2, but a few months before the Cholera. It also prevailed in the Atlantic States and in Europe.

Mr. Noah Webster, in his learned, but neglected work on Epidemic diseases, of which I hope he will give the profession a new and revised edition, has said much on the relation of influenza and scarlatina with pestilential epidemics. The history of the years 1831 and 32, in the western states, would certainly furnish many facts in support of his propositions. The connecting links among them, will probably never be revealed to us; but the facts which establish the reality of their combination deserve to be recorded. They should all be diligently studied, as to their modes of propagation; and thus be made, if possible, to throw light on each other's paths.

We constantly speak of the mysterious march of Cholera, but is not that of other epidemics equally wonderful? Let us take influenza as an example.

The late Epidemic catarrh commenced in the East, overspread Europe, crossed the Atlantic, and extended its march into the valley of the Mississippi—so did the Epidemic Cholera.

They both prevail in all states of weather; and are, therefore, independent of the sensible qualities of the atmosphere.

Epidemic Catarrh or Influenza, is an irritation of the mucous membrane of the ærial passages—Epidemic Cholera of the mucous membrane of the digestive passages. The former bears a striking resemblance to common or endemic catarrh, the latter to common, endemic cholera morbus.

Each of them is supposed by the people to be contagious; but their sudden appearance throughout a whole city, without the possibility of tracing up the first cases to importation, the indisposition in some slight degree, at least, of nearly the entire population of a place, their brief duration, and sudden disappearance—indicate an atmospheric origin; and, as these characteristics are common to both, they bring them under one law of propagation, and clearly indicate, that if the Influenza is not contagious, we are not at liberty without *positive* proofs, to believe in the contagiousness of Cholera.

It would not be difficult to trace out other analogies between these epidemics; but every reader can extend the parallel, and convince himself, that they differ chiefly in the rapidity of their progress, and the destruction of human life which they respectively occasion.

I am aware, that to bring Epidemic Cholera under the same laws of propagation as Epidemic Catarrh, is not an explanation, in reference to the former, unless we knew the mode in which the latter extends itself; but it is one step towards a knowledge of the latent causes of these epidemics, to show that they overrun the earth under the government of one system of laws.

### IV. Pathology.

It is not my desire to go extensively into this subject. We are ignorant of the part of the body on which the poison makes its impress, and of the nature of that impression; but the disturbance or impairment of the different functions may be observed and estimated. Reasoning from the phenomena which they exhibited, in those who were affected in Cincinnati, I would say, that the remote cause, was both depressing, and irritating in its effects. The majority of those who suffered, were evidently debilitated, in both the organs of animal and nutritive life. In some cases, it is true, inflammatory fever was developed; but this was the consequence of temperament or idiosyncracy; and might be compared to the exciting effects of digitalis, when administered to persons of a sanguine temperament, who had not been subjected to the loss of blood previous to its use. Such cases must have occurred to every experienced physician. Examples of Cholera attended with active inflammatory symptoms, were, in fact, exceptions to the rule: the majority displayed only the signs of nervous irritation. The consequence of this morbid condition of the nervous system were perceptible in nearly all the functions. The gastro-intestinal mucous membrane was in a state of irritation and morbid secretion; the muscular coat associated with it, was thrown into increased and irregular contraction; the liver was nearly paralized in its specific function, so that little or no bile was secreted; the kidneys, especially in the advanced stages of the disease, acted feebly or not at all; the pulmonary mucous membrane, in a few persons, was irritated into catarrhal secretion; the skin was deeply impaired, both in its calorific and perspiratory functions; the muscles of locomotion were irritable, and disposed to take on spasmodic action; the heart was enfeebled, and the vessels appeared to co-operate badly with it, in propelling the blood to the more distant parts of the body; lastly, the lungs did not properly decarbonize the blood. All these different functional disturbances, seemed to me to result in a great degree, from an overthrow of the great, presiding function of innervation, accompanied in a few persons with developed inflammation, especially in the mucous membrane of the stomach and bowels.

As the nervous system is, in fact, *one* great apparatus *modified* in its different parts, or what is the same thing, in the different organs, it does not seem to be very important for us to ascertain which part receives the blow in Cholera. The effect spreads rapidly throughout the whole of that universal tissue, and impairs of course the functions of the various organs where it is present.

Now whether the remote cause be applied to the skin, the mucous membrane of the stomach and bowels, the mucous membrane of the lungs, or being absorbed through that membrane, it act upon the blood or inner lining of the vessels, the effects on the functions may still be the same, and the means of correcting them the same.

From these views, two great indications of cure spontaneously arose. First, to allay the irritation of the nervous system; and, secondly, to restore the impaired or suspended secretions, and restrain those which were in excess. When these indications could be fulfilled, the patient was saved, when they could not, he died. If, however, his temperament and habit strongly predisposed him to inflammation, the first indication was replaced, by that diminishing plethora and subduing inflammotory action.

These brief, and, perhaps, superficial speculations, are applicable to the earlier stages of the disease, rather than that of collapse, or prostration of all the functions—a state concerning which I am not disposed to add any thing to the many volumes now before the profession. I have already expressed the opinion, that, much harm has resulted from directing too much thought and care upon this *closing* period of the disease, instead of that in which it is a mild and manageable complaint; and shall, therefore, ask the attention of the reader chiefly to the latter.

### V. Treatment.

The more common and regular cases of the Epidemic, presented three stages, each running into the other—these were *Diarrhœa*, *Cholera* and *Collapse*. The first was sometimes attended with cramps in small portions of the muscular system; the second with more extensive cramps, and, in many cases, violent spasms; the third was often without either. A failure in the functions of the skin and liver existed from the beginning, and increased through all the *stadia* of the disease. The rice-water discharges belonged to the second and third. The means of fulfilling, in the first stage, the two great indications of cure, laid down in the preceding section, were few and simple, but when employed in the proper time and manner, proved almost invariably successful. I proceed to enumerate them.

1. The first step was to confine the patient to his bed, or at least to a warm room. In many cases it was extremely difficult to accomplish this; and I have no doubt that its absurd and presumptous neglect, destroyed a great number who might have been saved. In my own practice, I made it a rule, to refuse prescribing for any one laboring under a diarrhœa, unless he would promise to observe this *sine qua non* to the success of any other prescription. To keep on one's feet, abroad, in the cool or damp atmosphere, with diarrhœa, and suspended action of the skin and liver, must always be injurious; but to do so when these symptoms arise from the poison, which produces Epidemic Cholera, is fatal. Believing that to be successful, we should begin with this disease, in the first stage, and that the most important prescription we can make is a warm bed, I cannot too strongly urge on all who may see these pages, and have yet to combat the enemy, the indispensible necessity of coercing their patients on this point.

2. On taking to his room or bed, it was of great service to the patient to use a general warm bath, or bathe his feet in warm water, rendered stimulating with salt or powdered mustard. If the functions of the skin were not *deeply* impaired, they were much improved by this measure.

3. As the patient's appetite was frequently unimpaired, it was necessary to prohibit its indulgence. Solid food was constantly injurious. Almost total abstinence was best; and what he took, was least offensive when it consisted of solutions of fecula and gelatin—such as thin arrow root jelly and weak broths.

4. A hot infusion of some diaphoretic simple, such as balm, sassafras, sage, or thoroughwort, the *Eupatorium perfoliatum* of the Botanists, one of our native plants, was of great service. The last is the most powerful sudorific of the whole; and was, therefore, well adapted to the object in view; but being a nauseant, when the infusion was strong or the stomach irritable, it sometimes produced a vomiting, that was mistaken for that attendant on the disease.

5. The aqueous solution of camphor, effected by the aid of magnesia, as proposed I believe by Dr. Parish, an eminent physician of Philadelphia, was found useful as a diaphoretic; but I met with no facts that went in the slightest degree to sustain the reputation of that medicine as a *specific* in any stage of Cholera.

6. These measures being adopted in reference chiefly to the state of the skin, it was necessary to institute others, calculated to quiet the bowels, diminish their watery secretions, and restore the suspended action of the liver. These ends were sometimes accomplished by a salt or mustard seed emetic; in other cases, by an active cathartic of calomel and jalap, or calomel and rhubarb, or pills of calomel and the resinous cathartics, which, if the patient were of a full habit, and the diarrhœa had not continued very long, were attended with excellent effects. Other cases were successfully treated with large doses of calomel alone, such as twenty or thirty grains, given at once, and worked off the next day, with infusion of senna, or rhubarb and magnesia, or castor oil. The sulphates of magnesia and soda, were generally rejected, as being apt to promote watery secretions. The practice, however, which was generally pursued by the physicians of the city, and by the people in numerous instances without the *immediate* advice of any medical man, after it was recommended in the newspapers, was to administer calomel and opium combined; and this practice, I am persuaded was far more successful than any other. The opium being pulverized, the compound was exhibited in powder. The proportion of these two ingredients was varied according to the judgment of the physician, to suit the age and condition of different patients. One grain of opium to ten of calomel, constituted a common or average dose, to be repeated every three, four, or six hours. Sometimes the proportion of calomel was doubled, so that the patient soon took, forty, sixty, eighty, or a hundred grains of that medicine; but this energy was required in violent and threatening cases only. Instead of increasing the dose of calomel, the physician, in many cases, diminished that of the opium to half a grain or less. This was advisable in all cases, attended with fullness of habit, inconsiderable diarrhœa, or peculiarity of constitution in reference to narcotics. In the last case, it was sometimes found advantageous, to substitute the sulphate or acetate of morphia for the opium itself.

By this compound of calomel and opium, or its narcotic principle, the two great indications of which I have spoken, were, in general, effectually fulfilled. It accomplished, in the average of cases, what neither of the medicines separately could have effected; though a number, for various reasons, required the opium to be omitted. In others, from the prejudices of the patient, or some other cause, the calomel was rejected; but I believe few were satisfied with the results of this practice, except that mineral was replaced by the blue pill, rhubarb, scammony, jalap, or some other active cathartic. In the midst of these diversities, it still remains true, that the powder of calomel and opium, was the most efficient means which could be employed, in the stage of the disease we are now considering. One of its first effects was to allay the general nervous irritation, tranquilize the feelings of the patient, diffuse a glow throughout his system, appease his cramps if any existed, and moderate or suspend the violent peristaltic action of his bowels. To these, succeeded other effects, of an auspicious kind; such as an abatement in the mordid secretions of the mucous membrane, a general perspiration, an equable distribution of the heat of the surface, and at length a restoration of the suspended or impaired biliary secretion, the consequence and sign of returning health in the portal viscera. Whenever the alvine discharges became bilious, the patient was considered as safe; though too early a discontinuance of the medicine, or indulgences in diet, or going prematurely abroad, not unfrequently reproduced the disease. In most cases, the calomel would at length operate as an aperient, but sometimes it became necessary to administer a more active cathartic, in consequence of an unusual torpor of the intestines from the action of the opium.

When I reflect on the beneficial fruits of this practice, in Cincinnati, I cannot but feel some astonishment and regret, that it should not have been pursued *every where*. Had calomel been more liberally employed in Paris, might not the mortality of its citizens have been less than it was? The timidity of the French practice in regard to this medicine, is certainly not the offspring of observation, so much as ancient prejudice. The phenomena of the Epidemic in the metropolis of France, seem to have been almost identical with those which it exhibited in this city, and why should they not be met by the same therapeutic agent? If the citizens of the two places had very different constitutions, the effects of the Epidemic poison would have been different; but this does not seem to have been the case, and we are at liberty, therefore, to suppose, that the effects of calomel in France would have been as beneficial as in Ohio. However this may be, I consider the fact fully and firmly established, that calomel should be the basis of the treatment of Cholera in the valley of the Mississippi.

7. Cases occasionally occurred, in which bloodletting, either general or local, was demanded in the first or diarrhœal stage; but they were comparatively few in number. The pulse served as a guide in these cases; but indications were likewise drawn from the state of the abdomen. When this was tender on pressure, or distended and hard, or colic-pains supervened, blood-letting was required and did much good. To many of these cases, cupping or leeching would undoubtedly have been well adapted, and the former was sometimes employed with advantage; but from the want of a professional cupper, and our entire destitution of leeches, topical blood-letting was not often an available remedy.

8. Emollient fomentations to the epigastric and umbilical regions, were attended with a soothing and happy effect on the stomach and bowels; and contributed to excite the skin, and, perhaps, also, the liver.

Such were the means by which our physicians, generally, combatted the disease in its first stage; and they were almost invariably successful. Could the necessity of having early recourse to them, have been rendered manifest to our whole population, but few would have perished; but so rapid was the spread of the Epidemic, and so inadequate the utmost individual exertion, that this important information, was by no means so extensively disseminated as it should have been. Had the Board of Health, the Sanatory Committee, and the Trustees of the Township, co-operating with individuals and the Cincinnati Medical Society, at an early period, devoted some part of their time, and the public moneys placed at their disposal, to the diffusion of this kind of knowledge, instead of directing their charities upon the dying and the dead, the amount of their benefactions would have been much greater, than it proved to be. It is better to preserve one man from serious illness, than to bury a dozen at the public expense.

The treatment of the second stage or that which exhibited the symptoms of cholera morbus, did not differ materially from that which the stage of diarrhœa required.

The patient thus affected, was of course in bed, and had no disposition to take food; but he was invariably restless, and while he continued in that state, perspiration could not be induced.

When he had fever the lancet was beneficial; and cupping over the abdomen, either with or without scarification, was of service.

A sinapism, a blister, or a piece of flannel dipped in hot oil of turpentine, bound over the epigastrium, often tranquillized the stomach.

When the vomiting had not continued very long, or had not been copious, the operation of a mustard or salt emetic, was often followed by a cessation of that sympton.

It is, especially, to this stage of the disease, that the saline treatment with the non-purgative salts, as recommended by Dr. Stevens of London, was adapted. It was but little employed, however, in Cincinnati; partly I presume, from his book not having been generally read, and partly from an unwillingness to depart from the prevailing plan of treatment. I directed it once in the diarrhœal stage, and it proved successful. In another case, attended with vomiting it was unavailing. Dr. Colby, I am informed, found it to succeed perfectly in one violent case. Dr. Richards has informed me, that he obtained advantage, in many cases of obstinate vomiting, from small and repeated doses of the sulphate of magnesia.

The hot diaphoretic infusions, so serviceable in the first stage, were promptly rejected and did harm in the second, at least, until the vomiting was subdued. On the other hand, the effervescing saline mixture, or the common portable soda powders; small draughts of very cold water; or little lumps of ice, as recommended by Broussais, were followed, in most cases, by an abatement of the gastric irritability and vomiting. It is to this stage of the malady and not that of collapse, that the refrigorant treatment seems to me to be adapted.

In some instances, the most alarming irritability of stomach was subdued by starch injections with laudanum. Dr. Rives mentioned to me a striking case of this kind.

But the chief reliance, at last, was on calomel and opium or calomel alone. To be successful, it was necessary to administer them, especially the last, in large doses, and in powder with sugar, so as to promote their rapid diffusion over the surface of the stomach. There is not, I presume, a physician, in Cincinnati, who cannot testify to the efficacy of this practice. It was worth every other therapeutic means, both external and internal. The most violent vomiting would cease, whenever the stomach could be brought under the influence of this compound, or of the calomel uncombined; and a speedy return of the suspended secretions of the liver and skin, generally, followed; after which the patient commonly recovered.

Trials of opium alone, were sometimes made, or of the salts of morphia, or of laudanum, with ammoniated alcohol, or sulphuric ether, or the aromatic tinctures; but, I think, they were not found so beneficial, in allaying the irritability of the stomach, as calomel and opium, or even calomel, only, which, indeed, often succeeded better than the compound.

When called in, immediately on the access of the vomiting, we could often succeed with these means in arresting the disease, but our failures were not a few; and if the vomiting were suffered to continue for some hours, the utmost effort was too often unavailing, and the disease passed into the third stage —that of Collapse.

Of the treatment in this stage, what can I say? Certainly nothing either new or satisfactory. In Cincinnati, as in all the earth besides, a great majority of those who sunk into collapse, inevitably perished. The methods adopted here were such as had been recommended, in places where the Epidemic had previously prevailed, but their effects were such, that one is at a loss to know, for what reason they *were* recommended.

I saw every practicable mode of inward and outward stimulation employed, with but little benefit. I should rather say, that all kinds of *stimulants* were *applied*, for in reality but little *stimulation*, was in general *excited*. The skin, it is true, was often reddened under our rubefacients. But no sympathies either continuous, contiguous, or remote, to quote the language of Mr. John Hunter, were, in many cases established; and the patient continued to sink as rapidly, perhaps, as if nothing had been done. There *were* cases, however, in which the descent of the vital powers was arrested by these means, and life apparently preserved.

The mustard emetic was often administered for this, and proved, I think, one of the best remedies. In one case, attended by Mr. Wood, one of my most ingenious pupils, I advised an emetic of opium and tartar emetic; it operated, but the patient died.

In this case and another, I advised injections of hot water, as practised by some of the Sunderland physicians; but was disappointed in their effects.

To a patient of Dr. Walker, one of our young physicians, who saw much of the disease, the cold affusion was applied, at my suggestion. When the buckets of water were dashed on, the patient shivered, groaned, and writhed under it, evincing a sensibility to its impress, but no glow of heat, or reaction of the heart, followed its application.

External heat appeared to be useful in many cases; but it was only when the patient was quiet that it could be successfully applied. I did not use the warm bath, nor the hot air or vapour bath. The latter was employed by some physicians; but, as far as I have been able to learn, with no very beneficial effect.

Blood-letting was employed in a number of cases, but seemed of little service.

Opium, I think, was generally found injurious, or, at least, useless, in this stage, whether given alone or in combination; and was, I believe, but little employed.

The administration of pellets of ice, on which Broussais seems almost exclusively to have relied, was, as far as I know, but little practised in this stage of the malady, partly, I presume, from a want of faith in the alledged results of that practice. For myself, I must say that the reports of its efficacy, in the practice of that celebrated physician, are somewhat incredible, and require confirmation. Regarding the disease as a gastro-enteritis, it is easy to perceive that much of preconceived opinion might have blended itself with the results of his clinical observations, and vitiated their correctness.

But *is* the disease an inflammation of the mucous membrane of the stomach and bowels? If so, it must be one of the most violent kind; and still for its cure, even Dr. Broussais does not venture to prescribe copious and repeated blood-letting; although that is the appropriate remedy for such an inflammation. Further, the same distinguished physician, after condemning the stimulant treatment in the stage of collapse, admits that it is better than *no* treatment; notwithstanding it is directly calculated to aggravate the inflammation! In all this there seems to be some mystery; and one cannot but suspect that this eminent pathologist, has found some difficulty in compelling this anomalous Epidemic to wear the shackles of his gastro-enteritic hypothesis. The opinion of the French Academy, that it is a catarrhal, rather than an inflammatory affection of the mucous membrane of the stomach and bowels, seems to quadrate much better with the facts.

I have been favoured with a letter from the respectable Dr. Henry Bronson, of Albany, written after he had witnessed many fatal cases of Cholera, both on the St. Lawrence and the Hudson, in which he observes, that a great number of different and *opposite* means have been found to rescue those who had sunk into collapse; and, that in attempting to determine what was common to the whole, in their *modus operandi*, one can discover nothing but that of their making a *strong impression on the system*—imparting to it, to speak figuratively, a kind of concussion, by which the torpid and sinking energies of the system are aroused, and the organs again put into action. I am disposed to think, that a more definite *rationale* of our treatment of this stage cannot be given in the present state of the sciences.

It must be admitted, however, that when, from a failure in the powers of the heart, the blood is not impelled to the distant parts of the body, but accumulates in the organs of the cranium, chest, and abdomen, or in some of them, that the phenomena of collapse will vary with the seat of the congestion; and that the means of cure should likewise vary. Thus, if the portal viscera be engorged, injections of hot water medicated, would be proper, and large doses of stimulating cathartics; or a mustard emetic; if the lungs and cavities of the heart are its special seat, blood-letting and antimonials might do good; and if the brain is specially engorged, as I saw in a patient of Dr. Woodward, who labored under profound apoplectic stupor, the loss of blood, in copious quantities, would seem to be the best, if not the only efficient remedy; but still, in that case it failed. The fact is, that those organic congestions, whether seated in the sinuses of the brain, the coronary and pulmonary vessels, or the portal circle, are but secondary effects; and if they could be removed by therapeutic efforts, it would by no means follow, that the patient would recover; for the debility of the circulatory apparatus, which occasioned them, might, and indeed generally does, remain, and continue to increase till the movements of the smitten and enfeebled heart entirely cease.

Of the consecutive fever, for a reason that will presently appear, I saw so little, that I will not venture to say any thing concerning it; except that nothing new, as far as I have heard, was employed by any of our physicians.

### V. Quackery in the Treatment.

It would be wrong to bring this history to a close, without referring to the Steam, or Thompsonian practice, as applied to the cure of the Epidemic. The physicians of the South know but little of this quackery from observation, but as an account of it was published in a former number of this Journal, they cannot be ignorant, that it consists chiefly in the administration of emetics of the *lobelia inflata*, and the subsequent use of concentrated tinctures of Cayenne pepper, myrrh and other stimulants, with the application of external heat, and a total abjuration of the lancet, opium, and calomel! Considered, therefore, in reference both to what it employs and what it rejects, it is a powerful practice, and must necessarily do either good or harm. That it sometimes did the former, cannot be denied; but I am satisfied that it much oftener perpetrated the latter. Indeed, I have heard of many cases in which it was manifestly prejudicial, and, firmly believe, that it was often fatal, where a discriminating and scientific practice might have succeeded. Still it has been boldly asserted, and unblushingly published to the world, that this practice was found, in Cincinnati, to be more successful than that of the regular members of the profession! Was the working with Thompson's patent, *confined* to illiterate men, who are too indolent to pursue an honest occupation for a livelihood, this notice would be less requisite; but when we discover among its advocates a number of respectable and intelligent citizens, such as members of the Bar and Trustees of our Medical College; when we see a regular Journal, established for sounding forth its praises, and find reputable booksellers, interested in the distribution of such a work, by which worthy, but ignorant and credulous people, are deluded into a false confidence, and the partition-wall between science and empyricism broken down; it is the duty of the former to assert its claims, and maintain its dignity. This is due, not merely to its own character, but to the interests of the community; and, as far as this Journal is able, will at all convenient times be done—whatever may be the apathy of some who ought to speak out; or the absurd preference of others, for a practice founded on no knowledge of the animal economy, over that, which rests on the collective experience of all ages and all nations.

### VI. Conclusion.

In concluding, I must again speak of myself. It is necessary to tell the distant reader (in this region every body knows the fact) that after having been abused for announcing the existence of the Epidemic, and warning the people to be on their guard, I was subsequently calumniated with the extremest virulence, and the greatest injustice. In all the surrounding country it was reported, that I saw but little of the Epidemic, for that, having lost *nearly* all my patients, I had, in fear and discouragement, retired from the scene of action, soon after the disease made its appearance; and feigning sickness, continued in seclusion till the danger was over.

I cannot suppose that any respectable member of the Profession, who may read these pages, will object to my stating a few facts, in contradiction to calumnies so unfounded, and so degrading to my professional and moral character.

I declare, then, that as far back as the 28th of September, I had a most violent case of Epidemic Cholera; but, as the patient recovered, I did not enumerate it among those which ushered in the Epidemic; and that until the 16th of October, a period of *nineteen days*, I was in the midst of those who laboured under it, giving incessant advice, both as an attending and a consulting physician. That from making almost daily publications on the means of prevention and cure, a great number of persons, whom I do not attend in ordinary, were attracted to me, thus affording me ample opportunities of seeing the disease in all its varieties; that throughout the whole, my exertions, both of body and mind, were unremitting; that I got but little sleep at night, or rest by day, but was kept in a state of continual action and excitement; that these causes, at length produced an inflammatory affection of the brain, from which I had suffered several times before, which required the loss of two quarts of blood, under the direction of several respectable medical friends, and confined me till in November; that from the beginning, it was attended with a few symptoms of the Epidemic, as was, indeed, every kind of illness, and that in its decline, these became more numerous, and protracted my confinement till the Epidemic had ceased. Now, during the whole period of nineteen days, through the last ten of which the disease was extensively and fatally prevalent, as the bills of mortality have shown, I lost but *three* patients. One of these sunk down in his yard, having neglected the early symptoms, and I saw him but *once*, from the great press of earlier engagements. The second was a gentleman 76 years of age, who had laboured under chronic diarrhœa from February, and had, twice during the summer, been expected to die of that malady. He was, of course one of the first attacked, but got so well as to go abroad. From indulgences in diet and imprudent exposure, he relapsed. He refused to take calomel; and, indeed, anticipating that he would become a victim to the Epidemic, was averse to medical assistance, and expired without being subjected to the treatment which, at that time, I was, both in private and public, recommending to others. The third and last patient whom I lost, was a lady, in whom the disease appeared in the form of hysteria, succeeded by fever, without any of the ordinary symptoms of Cholera. I saw her before day, on Sunday morning, and not, again, till Monday forenoon, when she still had no symptoms of the Epidemic. At eleven the following night, I was summoned to her a third time, and found that vomiting, coldness, and collapse, had suddenly supervened, and she expired early the next morning. She was visited, oftener than I visited her, by another gentleman, whose extensive practice in the Epidemic was at least as successful as that of any other physician in the city. THESE ARE ALL THE PATIENTS I LOST; IF THERE ARE ANY OTHERS THEY CAN BE POINTED OUT BY MY TRADUCERS.

*Cincinnati, December* 1*st*, 1832.

